# Distinct shed microvesicle and exosome microRNA signatures reveal diagnostic markers for colorectal cancer

**DOI:** 10.1371/journal.pone.0210003

**Published:** 2019-01-04

**Authors:** Maoshan Chen, Rong Xu, Alin Rai, Wittaya Suwakulsiri, Keiichi Izumikawa, Hideaki Ishikawa, David W. Greening, Nobuhiro Takahashi, Richard J. Simpson

**Affiliations:** 1 Department of Biochemistry and Genetics, La Trobe Institute for Molecular Science (LIMS), La Trobe University, Melbourne, Australia; 2 Department of Applied Biological Science, Graduate School of Agriculture, and Global Innovation Research Organisation, Tokyo University of Agriculture and Technology, Tokyo Japan; University of South Alabama Mitchell Cancer Institute, UNITED STATES

## Abstract

Extracellular vesicle (EV) microRNAs are of major interest as potential diagnostic biomarkers in all cancer types. This study aims to identify miRNA profiles of shed microvesicles (sMVs) and exosomes (Exos) secreted from the isogenic colorectal cancer (CRC) cell lines SW480 and SW620 and evaluate their ability to predict CRC. Deep sequencing of miRNAs in parental cell lysates (CLs) and highly-purified sMVs and Exos was performed. We focused on miRNAs enriched in EVs and dysregulated miRNAs in metastatic cells (SW620) relative to primary cancer cells (SW480). We investigated the ability of EV miRNA signatures to predict CRC tumours using 594 tumours (representing different pathological stages) and 11 normal samples obtained from TCGA. In SW480 and SW620 cells we identified 345 miRNAs, of which 61 and 73 were upregulated and downregulated in SW620-CLs compared to SW480-CLs, respectively. Selective distribution of cellular miRNAs into EVs results in distinct miRNA signatures for sMVs and Exos in each cell line. Cross cell line comparisons of EV miRNA profiles reveal a subset of miRNAs critical in CRC progression from primary carcinoma to metastasis. Many miRNAs non-detectable (<5 TPM) in CLs were significantly enriched (>1000 TPM) in secreted EVs. Strikingly, *miR-7641* which is non-detectable in SW480-CL but upregulated in SW620-CL is highly enriched in EVs secreted from both cell lines. Pearson correlation analysis demonstrated that EV miRNA profiles can be used to predict CRC tumours with ~96% accuracy. Our findings suggest that EV miRNA profiles from CRC cell lines may allow prediction of CRC tumours, and that *miR-7641* may serve as an attractive candidate for the specific, non-invasive diagnosis and prognosis of CRC.

## Introduction

Extracellular vesicles (EVs) are nano-membranous particles (30–2000 nm) released by most cell types and function in cell-cell communications [1, 2]. According to particle size, two major EV subtypes have been reported: shed microvesicles (sMVs), also referred to as microparticles or microvesicles that range in size from 400–1500 nm and exosomes (Exos) that range in size from 50–150 nm [3]. Exosomes are released as intraluminal vesicles (ILVs) from the multivesicular bodies (MVBs), which are formed by budding of the limiting membrane of late endosomes [1]. By contrast, sMVs are generated by the direct budding and fission of the plasma membrane [4]. To date, EVs have been found to be secreted not only by eukaryotic cells but also from plant cells and pathogens (e.g., bacteria, mycobacteria, archaea, and fungi) [5, 6].

During EV biogenesis, cellular bioactive cargo molecules such as protein, lipid, DNA and RNA are selectively packaged into the EVs, [1]. Comprehensive studies have identified ALIX, TSG101, RAB27A, RAB11B, CD9, CD63, CD81 and CD82 as stereotypic markers for exosomes and KIF23 as for sMVs [7, 8]. Since Valadi and colleagues reported ~121 microRNAs (miRNAs), small noncoding RNAs (~ 22 nt) that function in targeting mRNAs for cleavage or translational repression in animals and plants [9, 10], EV-associated miRNAs have attracted much attention because of their multiple functions in cellular activities and, importantly, their potential as diagnostic and prognostic biomarkers for cancer [11, 12]. For example, miR-200 promotes epithelial-to-mesenchymal transition (EMT) and breast cancer cell metastasis through the exosomal transfer into non-metastatic cells [13]. Lung and pancreatic tumour-derived EVs have been reported to transfer miR-21, activate TLR7 receptor on murine myoblasts and promote apoptosis through c-Jun N-terminal kinase (JNK) activity in C2C12 immortalized myoblasts or primary myoblasts [14]. Because EVs from a variety of cancer types are found in circulating body fluids such as blood their miRNA signatures are being extensively studied as potential diagnostic/ prognostic markers of disease [15].

Colorectal cancer (CRC), a leading cause of cancer death in the Western world [16], is the third most commonly diagnosed cancer in males and the second in females, with an estimated 1.4 million new cases and 50,630 deaths occurring in 2018 [17]. Metastatic CRC is the principal cause of death and only early diagnosis can improve the treatment. Some groups have demonstrated that circulating miRNAs can be a powerful tool to predict early CRC. Chen *et al*. reported that miRNAs, such as miR-134, miR-146a, miR-221, miR-222 and miR-23a, were deregulated in the serum of CRC patients compared to healthy controls [18]. Huang and colleagues identified that plasma miR-29a and miR-92a have significant diagnostic value for advanced colorectal neoplasia [19]. Some studies have also identified miRNA candidates for early CRC diagnosis in circulating EVs. For example, seven miRNAs (let-7a, miR-1229, miR-1246, miR-150, miR-21, miR-223, and miR-23a) were significantly higher in serum exosomes of primary CRC patients, compared to healthy controls [20]. Plasma extracellular RNA profiles identified that six EV RNAs including five miRNAs (miR-1343-3p, miR-125a-5p, miR-708-5p, miR-381-3p and miR-543) can differentiate CRC patients from healthy controls with an area under curve (AUC) of 0.76 (sensitivity = 0.72 and specificity = 0.80) [21]. Further, plasma exosomal miR-125a-3p and miR-320c were up-regulated in early stage CRC patients (stage I and II) and they showed significant correlation with nerve infiltration (P < 0.01), but not with tumour size, infiltration depth, and differentiation degree (P > 0.05) [22].

Recently, we described the isolation of two populations of exosomes (A33-Exos and EpCAM-Exos) as well as sMVs from the CRC cell line LIM1863 and identified 254 cellular miRNAs [23] of which 63 selectively distribute to the EVs–thereby, providing miRNA signatures for these EV subtypes. Interestingly, of 32 miRNAs that selectively distribute to A33-Exos, 13 have not been previously reported in publicly-accessible miRNA databases for human CRC tissue/biofluids [23]. In addition, we identified 2,389 mRNAs, 317 pseudogene transcripts, 1,028 long noncoding RNAs and 206 short non-coding RNAs selectively distributed to LIM1863 EVs, of which several RNA species are up-regulated in tumour biopsies [24]. Here, using RNA-Seq analysis we compared the miRNA profiles of EVs (Exos and sMVs) secreted from two isogenic CRC cell lines–primary carcinoma-derived SW480 cells and its lymph node metastatic variant SW620 cells [25]. We sought to compare the miRNA profiles of EVs secreted from SW480/SW620 cells and ascertain whether they reveal a subset of miRNAs critical in the aetiology of CRC and provide potential markers of the disease. Because there is increasing evidence that cancer cell EVs are present in biofluids such as blood, we also sought to investigate whether CRC cell line derived EV-miRNA profiles might provide a useful tool to predict CRC tumours.

## Material and methods

### Cell culture and exosome isolation

Human CRC cell lines SW480 (CCL-228) and SW620 (CCL-227) were from ATCC (Manassas, VA, USA). Cells were initially cultured in 175-cm^2^ flasks in RPMI-1640 medium (Life Technologies, CA) supplemented with 10% (v/v) foetal bovine serum (Thermo Fisher Scientific, CA) and 1% (v/v) Penicillin Streptomycin (Pen/Strep, Thermo Fisher Scientific) at 37°C and 10% CO_2_ and then transferred to CELLine AD-1000 Bioreactor Flasks (Integra Biosciences, NH) and continuous culture performed over a period of several weeks, as described [26]. Cell culture medium (CM) from two separate bioreactor flasks (150 ml) was collected for EV isolation (in duplicate, biological replicates), as described [26]. Briefly, low-speed centrifugation removed cells and cell debris. Then the supernatant was centrifuged at 10,000 × g for 30 min to obtain shed microvesicle (sMVs). The sMV-depleted supernatant was further centrifuged at 100,000 × g for 1 h to isolate crude exosomes. OptiPrep density gradient (100,000 × g 18 h) was employed to obtain purified exosomes. Protein quantification was performed by protein staining (SYPRO Ruby) densitometry following 1D-SDS-PAGE [26].

### Cell lysate preparation

Cell lysates were prepared as previously described [26]. Briefly, SW480 and SW620 cells were cultured in 10-cm dishes to 70% confluency. The growth medium was removed and TRIzol reagent (1 mL) added to lyse the cells and the lysate subjected to RNA extraction.

### Cryo-electron microscopy

Cryo-EM imaging of EV preparations was performed essentially as described [27, 28], with minor modifications. Briefly, EV preparations (~2 μg protein, non-frozen samples prepared within 2 days of analysis) were transferred onto glow-discharged C-flat holey carbon grids (ProSciTech Pty Ltd). Excess liquid was blotted and grids were plunge-frozen in liquid ethane. Grids were mounted in a Gatan cryoholder (Gatan, Inc., Warrendale, PA, USA) in liquid nitrogen. Images were acquired at 300 kV using a Tecnai G2 F30 (FEI, Eidhoven, NL) in low dose mode. Size distribution of vesicles (range 30–>1000 nm) was calculated using ImageJ for the 12 fields of view (~200 different vesicles for each EV preparation).

### Nanoparticle Tracking analysis

EV diameter (size) and concentration was determined using the NanoSight NS300 system (Malvern, UK) equipped with a blue laser (488 nm). Briefly, EVs were diluted in water (~8 × 10^8^ particles/ml) and loaded into a flow-cell top plate using a syringe pump. Three videos (1 min) were recorded for each sample and analysed by NTA software (Build 3.1.45).

### Western blotting

Western blot analyses of cell/EV samples (10 μg protein) were performed as described [26]. Briefly, membranes were probed with primary mouse anti-TSG101 (1:500, BD Biosciences, San Jose, CA), mouse anti-Alix (1:1000, Cell Signalling Technology Danvers, MA), mouse anti-CD9 antibody (1:1000, Abcam, Cambridge, MA), mouse anti-KIF23 (1:1000, Thermo Fisher Scientific, CA), and rabbit anti-GAPDH (1:1000, Abcam, Cambridge, UK) in TTBS (Tris buffered-saline containing 0.1% Tween-20) for 1 h. After washing with TTBS (3 × 10 min), membranes were probed with secondary antibody (IRDye 800 goat anti-mouse or IRDye 700 goat anti-rabbit IgG, 1: 15,000). The fluorescent signals were detected using the Odyssey Infrared Imaging System, v3.0 (Li-COR Biosciences, Nebraska, USA).

### Total RNA isolation

Total RNA extraction from samples was performed (in duplicate), as described previously [24].

### Small RNA library construction and deep sequencing

Small RNA (18~30 nt) libraries for SW480/SW620 cells and derived EVs (sMVs and exosomes) were constructed using Illumina TruSeq Small RNA Sample Preparation Kit v2 and sequenced on a HiSeq 2000 platform (Illumina), according to the manufacturer’s protocols. Briefly, small RNAs (18~30 nt) were fractioned on a 15% Tris-borate-EDTA (TBE) polyacrylamide gel (Life Technologies, CA) from total RNA (200 ng), purified by centrifugation, and ligated with adaptors. Small RNAs were then reverse transcribed into cDNAs and amplified using the adaptor primers for 14 cycles. The cDNA fragments (~150 bp) were isolated from a 6% TBE PAGE-gel and directly used for cluster generation by using TruSeq PE Cluster Kit v3 (Illumina). Using TruSeq SBS Kit v3 (Illumina) biological replicates (n = 2) were sequenced for each sample.

### Small RNA annotation

Clean reads were obtained for subsequent analyses by removing low-quality, adapter, and short (<18 nt) reads from the raw reads. Clean reads were first aligned to the human reference genome (GRCh38, http://hgdownload.soe.ucsc.edu/goldenPath/hg38/bigZips/) by SOAP2 [29] without mismatch. Then, miRNAs were identified and profiled by mapping the clean reads to human miRNA precursors obtained from miRBase (v21, http://www.mirbase.org). Other noncoding RNA types (rRNA, tRNA, snRNA, snoRNA, srcRNA, srpRNA) in the clean reads were annotated using Rfam and GenBank databases by BLAST software [30]. Repeat-associated small RNAs and mRNA degraded fragments were characterized by mapping clean reads to repeat, exon and intron regions on the human genome. Target prediction and pathway analyses were performed by using Ingenuity Pathway Analysis (IPA) [31].

### qRT-PCR validation

Quantitative real-time PCR (qRT-PCR) was used to validate expression levels of 11 candidate miRNAs (let-7a-5p, miR-10a-5p, miR-182-5p, miR-183-5p, miR-192-5p, miR-193b-3p, miR-19b-3p, miR-222-5p, miR-28-3p, miR-339-5p and miR-486-5p) and a housekeeping gene RNU43 was used as the internal control. Briefly, total RNA (100 ng) from cells/ derived-EVs was obtained as described above, transcribed into cDNAs by reverse transcription (RT) primers using iScript Reverse Transcription Supermix for RT-qPCR (Bio-Rad Laboratories Inc., USA). Then, cDNA templates (2 μl), forward and universal reverse primers (2 μl), 1×SsoAdvanced Universal SYBR Green Supermix (10 μl, Bio-Rad Laboratories Inc., USA) and ddH_2_O (6 μl) were mixed together to make a super qRT-PCR mix (final concentration of primers: 0.3 μM). Three amplifications for every candidate miRNA in each sample were run on a CFX96 Touch Real-Time PCR Detection System (Bio-Rad Laboratories Inc., USA). All miRNA expression levels were normalized to RNU43 using CFX Manager Software v3.1 (Bio-Rad Laboratories Inc., USA).

### Clinical sample miRNA data

Clinical sample miRNA data used in this study were obtained from The Cancer Genome Atlas (TCGA). miRNA expression profile data for normal and CRC tumour tissues were obtained from two CRC projects (TCGA-COAD and TCGA-READ) of TCGA, which recruited miRNA expression profiles of 11 normal colon tissues, and CRC tumours of known pathological stage—103 stage-I, 223 stage-II, 179 stage-III, and 89 stage-IV tumour tissues. Original miRNA counts, and normalized miRNA expression files were downloaded for subsequent analysis.

We also obtained serum and plasma exosomal miRNA profiles of CRC patients from two publicly-available datasets [20, 21]. The serum exosomal miRNA study screened 88 primary CRC patients and 11 healthy controls (HCs) using microarray technology and identified 69 miRNAs that were up-regulated in serum-derived exosomes from CRC patients relative to serum-derived exosomes from healthy controls. The plasma exosomal miRNA study recruited a total of 159 CRC patients from different pathological stages (58 stage I, 46 stage II, 28 stage III and 27 stage IV) and HCs (n = 50).

### Statistical analysis

edgeR [32] was used to identify miRNAs enriched in the EVs and dysregulated in the tumour samples in comparison with normal tissues. We first filtered miRNAs expressed no more than 5 transcripts per million reads (TPM). Raw miRNA read counts were then used in edgeR as input for differential expression analysis. Strict criteria were used to select differentially-expressed miRNAs as follows: log2 fold change (log2FC) >1 (for up-regulated miRNAs) or log2FC <-1 (for down-regulated miRNAs), p-value <0.05, and false discovery rate (FDR) <0.05. The Pearson correlation coefficient calculated by ‘cor’ (an R package) was used to evaluate the correlation between cell/EVs and clinical samples.

## Results

### Extracellular vesicle isolation, purification and characterization

**Fig 1:**
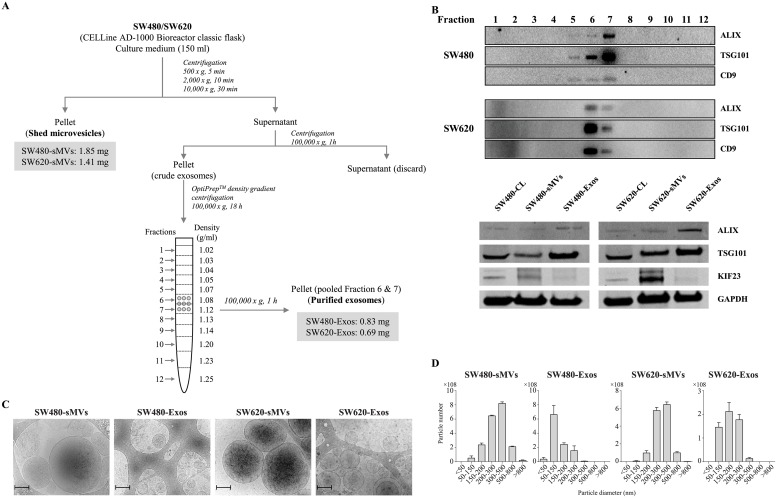
Purification and characterization of sMVs and exosomes secreted from human colorectal cancer cell lines SW480 and SW620. **A**, A combination of differential centrifugation and density gradient ultracentrifugation (OptiPrep) was used to purify shed microvesicles (sMVs) and exosomes (Exos) in high yield. **B**, Western blot analysis of EV markers. Antibodies for stereotypic exosomal markers (ALIX, TSG101, and CD9) revealed enrichment of exosomes in fractions 6–7 (buoyant density, 1.08–1.12 g/mL) (up panel); antibodies of TSG101, ALIX, KIF23 and GAPDH showed KIF23 enriched in sMVs (below panel). **C**, Cryo-electron microscopy was used to determine median particle diameters of purified sMVs and Exos: SW480-sMVs 266 nm, SW480-Exos—119 nm, SW620-sMVs—486 nm, and SW620-Exos—127 nm (scale bar: 200 nm). Images representative of 2 biological replicates with n = 12 images obtained for each replicate; vesicle diameters determined using ImageJ software. **D**, NanoSight Tracking Analysis was used to calculate size distribution of sMVs and exosomes: SW480-sMVs, 339 nm; SW480-Exos, 138 nm; SW620-sMVs, 327 nm; SW620-Exos, 201 nm (representative of a single biological replicate, with 3 technical replicates performed, and data averaged and merged).

SW480 and SW620 cells were characterized as previously described [26]. Cells were maintained and cultured in two CELLine AD-1000 Bioreactor classic flasks and 10 collections of culture media (total volume 150 ml) were made over a period of 5 days. As shown in Fig 1A, shed microvesicles (sMVs) and crude exosomes were isolated from the CM by sequential centrifugation. The presence of sMVs in the 10,000 × g pellet was confirmed by the stereotypic sMV marker KIF23 [8] (Fig 1B). The yields of SW480- and SW620-derived sMVs were 1.85 mg and 1.41 mg protein, respectively. Next, the crude exosomes (reconstituted in 500 μl PBS) were further purified by density gradient centrifugation using a 5–40% iodixanol (OptiPrep) density gradient. Exosomes were enriched in factions 6 and 7 (buoyant density 1.08–1.12 μg/ml) based on western blot analysis of the stereotypic exosomal marker proteins Alix, TSG101 and CD9 (Fig 1B). The yields of SW480- and SW620-derived exosomes were 0.83 mg and 0.69 mg protein, respectively. Both cryo-EM and NTA analyses (Fig 1C and 1D) showed the particle size of SW480 and SW620 exosomes to be in the range 40–200 nm (mean size: SW480–119 nm and SW620–127 nm), while those of sMVs were in the range 50–1500 nm (mean size: SW480–266 nm and SW620–486 nm). Total RNA was extracted from SW480 and SW620 cell lysates and their purified EVs using Trizol reagent (Life Technologies, CA). RNA quality analysis, evaluated using an Agilent 2100 Bioanalyzer (S1 Fig), revealed that small RNAs (<50 nt) were enriched in the EVs.

### Differential expression of miRNAs in SW480 and SW620 cell lysates

**Fig 2:**
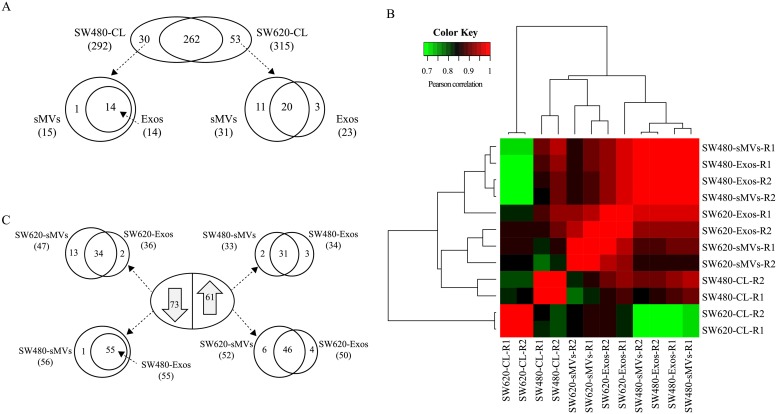
Analysis of cellular miRNAs in human colon cancer cell models. **A**, Venn diagrams of miRNAs identified in SW480/ SW620 cell lysates (biological replicates combined) and for each cell line the number of unique miRNAs that selectively distribute to secreted EVs: sMVs and Exos. **B**, Correlation coefficient heat map shows limited variability between biological replicates (R1-2) and illustrates differences between miRNA profiles of SW480/SW620-CLs and their secreted EV subtypes. **C**, Differential miRNA expression analysis (log_2_ FC >1 or <-1, p-value <0.05, FDR <0.05) in SW480/SW620 cell lines by edgeR revealed 134 dysregulated miRNAs (S2 Table). Of these, 73 are miRNAs down-regulated in SW620-CL and 61 up-regulated, relative to SW480-CL; the Venn diagrams show the number of dysregulated miRNAs that selectively distribute to SW480/SW620 secreted EVs.

Small RNA sequencing was performed to profile miRNA expression in SW480 and SW620 cell lysates (CLs) and their released EVs. Initially, a total of 160.46 million raw reads were generated by an Illumina HiSeq-2000 system for all the samples, yielding an average 12.77 million clean reads. Clean reads were aligned to miRBase (v21) to profile known human miRNAs in each sample. We used total miRNA mapped reads for normalization purposes, and then filtered lowly-expressed miRNAs (<5 TPM) to obtain a total of 345 miRNAs in SW480 and SW620 CLs (S1 Table); 292 and 315 being identified in SW480 and SW620 CLs, respectively. Among these, 102 (29.6%) were identified as passenger (star) miRNAs and 262 cellular miRNAs are common to both cell lines (Fig 2A). A heat map (Fig 2B) of sample correlations, based on their miRNA profiles, indicated sMVs, exosomes and cells contain distinct miRNA signatures (clusters); 30 and 53 miRNAs were found to be present exclusively in SW480 and SW620 CLs, respectively. Of these, the most abundant miRNAs found uniquely in SW480 CLs included *miR-371a-5p*, *miR372-3p*, *miR-373-3p*, and *miR-509-3p*. In the case of SW620 CLs, *miR-1224-5p* and *miR-125b-5p* were the most abundant.

Next, the statistical software package edgeR was used to identify miRNAs differentially expressed in SW480/ SW620 CLs. We identified 134 dysregulated miRNAs (Fig 2C, S2 Table). Of these, 61 SW480 cellular miRNAs were found to be upregulated in SW620 CLs, and 73 downregulated. The most highly-dysregulated miRNAs in SW480/SW620 CLs are listed in Table 1. Most prominent of these are four miRNAs in SW480 CLs (*miR-371a-5p*, *miR-509-3p*, *miR-200c-3p*, and *miR-141-3p*) whose levels are >28-fold higher when compared with SW620 CLs and 6 members of the miR-17~92a cluster (*miR-17-5p*, *miR-18a-5p*, *miR-19a/b-3p*, *miR-20a-5p*, and *miR-92a-1-5p*) that are significantly upregulated in SW620 relative to SW480 CLs (Table 1, S2 Table). Using qRT-PCR, and RNU43 as the internal control, we validated 11 miRNAs in all samples. Then, we normalized the miRNA expression by using SW480-CL as the control. Normalized expression by both qRT-PCR and miRNA sequencing revealed 69.7% of the comparisons to be consistent (S2 Fig). IPA analysis revealed cellular dysregulated miRNAs were involved in the pathways associated with CRC progression and metastasis, such as ‘colorectal cancer metastasis signaling’, ‘p53 signaling’ and ‘Wnt/β-catenin signaling’ (S3 Fig). Experimentally validated target genes of the cellular dysregulated miRNAs revealed known oncogenes and tumour suppressors from these three pathways, including APC, TP53, KRAS, CD44 and TGFBR2 (S3 Fig).

**Table 1:** Selected miRNAs highly expressed in SW480/SW620 cell lines.

Cell line	miRNA^a^	SW480^b^	SW620^b^	Log_2_FC^c^	TCGA^d^
SW480	miR-371a-5p*	247.74	0.13	-11.277	
	miR-34c-5p	27.3135	0	-11.13	Down
	miR-342-5p	12.7025	0	-10.021	Down
	miR-342-3p	12.3445	0	-9.977	Down
	miR-372-3p	100.0815	0.134	-9.966	
	miR-509-3p	317.6	0.71	-9.346	
	miR-373-3p	119.141	0.2615	-9.319	
	miR-200c-3p	205.48	9.88	-4.953	Down
	miR-141-3p	1697.01	88.54	-4.842	Up
	miR-203a-3p	619.21	100.72	-3.198	Up
	miR-424-3p*	558.24	133.38	-2.639	
	miR-450b-5p	211.02	60.68	-2.373	Up
	let-7e-5p	4865.66	2220.8	-1.701	Down
	miR-10a-5p	322652.89	161525.68	-1.585	Up
	miR-146b-5p	292.59	146.26	-1.581	Up
	miR-125a-5p	1741.36	881.99	-1.568	Down
	miR-26a-5p	20625.27	10740.42	-1.524	Up
	miR-29a-3p	2196.02	1204.33	-1.445	Up
	miR-423-3p	6145.78	3559.41	-1.366	Down
	miR-21-5p	109349.32	65249.43	-1.327	Up
	miR-96-5p	207.37	131.89	-1.238	Up
	miR-22-3p	15892.02	10135.87	-1.23	Up
	miR-182-5p	82988.6	53721.39	-1.214	Up
	miR-378a-3p	6214.12	4003.12	-1.214	Down
	miR-500a-3p*	979.22	673.06	-1.125	Down
SW620	miR-142-5p	37.23	3579.56	6.006	Up
	miR-142-3p	7.89	498.91	5.395	Up
	miR-181a-3p	17.33	986.11	5.251	Up
	miR-375	626.96	29844.77	4.989	Down
	miR-192-5p	3291.59	111573.13	4.502	Up
	miR-194-5p	50.2	1538.3	4.358	Up
	miR-1224-5p	3.2065	60.325	3.648	
	miR-100-5p	125	2267.91	3.601	
	miR-1247-3p*	30.66	450.09	3.296	Down
	miR-125b-5p	4.009	56.979	3.246	Down
	miR-181a-5p	6098.54	60778.94	2.735	Down
	miR-140-3p	145.57	1190.55	2.45	Down
	miR-181b-5p	1108.63	8695.7	2.387	Down
	miR-151a-5p	474.61	2362.55	1.735	Up
	miR-19a-3p	141.13	652.83	1.629	Up
	miR-19b-3p	481.3	2171.18	1.591	Up
	miR-92a-1-5p	110.98	460.75	1.469	Down
	miR-582-3p	144.06	593.63	1.459	Up
	miR-151a-3p	1035.92	4226.32	1.447	Up
	miR-196a-5p	212.3	858.05	1.431	Up
	miR-486-5p	199.2	676.53	1.182	Down
	miR-17-5p	190.82	643.33	1.172	Up

* are passenger miRNAs.

^a^miRNAs annotated with superscript

^b^Average of normalized expression values from both biological replicates, >5 TPM observed in at least one replicate.

^c^p-value<0.05 and FDR <0.05.

^d^miRNAs dysregulated in CRC tumors compared to normal colon tissues (source, TCGA) according to dbDEMC 2.0; UP–upregulated, DOWN–down regulated.

### Cellular miRNAs selectively traffic into sMVs and exosomes

Next, we asked how many of the unique, as well as dysregulated, miRNAs found in SW480/ SW620 CLs sort into their cognate EVs. Of the 30 unique miRNAs found in SW480-CLs, 14 distribute to both sMVs and Exos while an additional miRNA (*miR-1262*) uniquely distributes to sMVs. In the case of the 53 unique SW620 CL miRNAs, 31 and 23 were found to distribute into sMVs and Exos, respectively; 20 of these sorts to both EV subtypes (Fig 2A). Concerning those miRNAs in SW480-CL whose expression levels are dysregulated (relative to their expression in SW620-CL), 55/73 of the up-regulated miRNAs are common to both SW480-derived sMVs /Exos, while 46/61 of the down-regulated miRNAs in SW480-CL distribute to both SW620-derived EV subtypes; interestingly, 6 and 4 of the up-regulated miRNAs in SW620-CL were observed to uniquely distribute to SW620-derived sMVs and Exos, respectively (Fig 2C).

### Cross cell line comparison of miRNA profiles in sMVs and Exos

In addition to comparing miRNA profiles of SW480/SW620 CLs, a further goal of this study was to compare the miRNA profiles of EVs derived from these isogenic cell lines to ascertain whether they reveal a subset of miRNAs critical in CRC progression from primary carcinoma (SW480 cell line surrogate) to metastasis (SW620 cell line surrogate)–information that might be used as markers to assist in the clinical staging of the disease. To address this question, we performed a cross cell-line comparison of miRNA profiles of the two major EV subtypes isolated from SW480/SW620 cell culture media.

**Fig 3:**
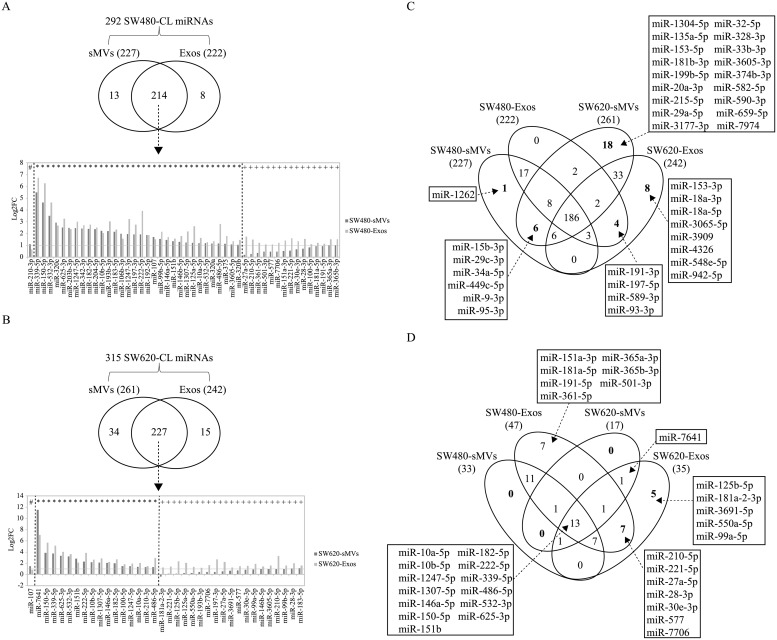
Selective packaging of SW480/SW620 cellular miRNAs into sMVs and exosomes. **A**, Of the 292 total SW480 cellular miRNAs detected, 227 and 222 distribute to sMVs and Exos, respectively (based on normalized expression >5 TPM); 214 miRNAs are common to both SW480-sMVs and–Exos, of which 48 are enriched according to differential miRNA expression analysis by edgeR (log2FC > 1, p-value <0.05, FDR <0.05); the bar plot below lists 32 common to sMVs and Exos (*), and 1 (#) and 15 (+) miRNAs enriched in sMVs and Exos, respectively. **B**, Of the 315 detected SW620 cellular miRNAs, 261 and 242 were distributed into sMVs and exosomes, respectively (based on normalized expression >5 TPM). 227 cellular miRNAs are common to both sMVs and exosomes. Differential miRNA expression analysis by edgeR (log2FC > 1, p-value <0.05, FDR <0.05) revealed 36 cellular miRNAs are selectively enriched in both EV subtypes; the bar plot below lists 16 miRNAs common to both EV types (*), and 1 (#) and 19 (+) miRNAs selectively enriched in sMVs and Exos, respectively. **C**, Venn diagram showing distribution of 186 miRNAs (S7 Table) that are common to all SW480/SW620-secreted EVs into their respective cell line-derived sMVs and Exos (S3–S6 Tables) and miRNAs identified specifically in each EV subtype. **D**, Venn diagram showing distribution of 13 significantly enriched (log2FC > 1, p-value <0.05, FDR <0.05) miRNAs in all SW480/SW620 EV types (S3–S6 Tables) and miRNAs enriched specifically in each EV subtype.

Of the 292 miRNAs (expression levels >5 TPM) in SW480-CL, 227 and 222 were detected in derived sMVs and Exos, respectively (Fig 3A, S3 and S4 Tables). 214 of these EV miRNAs are common to both EV subtypes while 13 and 8 are exclusively detected in derived sMVs and Exos, respectively. edgeR analysis of the 214 common miRNAs revealed 32 /214 to be significantly (log2FC>1) enriched in both EV subtypes; one (*miR-210-3p*) and 15 miRNAs (including the passenger miRNA of *miR-210*, miR-210-5p) are selectively enriched in SW480-sMVs and -Exos, respectively. Next, we applied this analysis to the SW620 cell model. Of the identified 315 SW620-CL miRNAs, a combined total of 276 miRNAs are found in SW620-sMVs (261, S5 Table) and SW620-Exos (242, S6 Table). 227/276 of these miRNAs are common to both EV subtypes; 34 and 15 are unique to SW620-sMVs and -Exos, respectively (Fig 3B). 16/227 miRNAs were significantly enriched in both SW620-sMVs/ -Exos (middle panel of bar chart); while one (left panel of bar chart) and 19 (right panel of bar chart) were enriched only in SW620-sMVs and SW620-Exos, respectively.

Next, we performed a cross cell-line (SW480/SW620) analysis of dysregulated sMVs and Exos miRNAs (see 4-way Venn diagram Fig 3C). It can be seen that a total of 186 miRNAs are common to all sMVs and Exos from these two cell lines (S7 Table). Interestingly, with the exception of miR-365a-3p and miR-365b-3p, 17 miRNAs observed in SW480 EVs are down-regulated in SW620-CLs, relative to SW480-CLs. In the case of the 33 miRNAs detected in SW620 EVs (S8 Table), including 17 SW620-CL up-regulated miRNAs; in addition, 1 (miR-1262), 18 and 8 miRNAs are found exclusively in SW620-sMVs and SW620-Exos, respectively. 6 miRNAs (miR-15b-3p, miR-29c-3p, miR-34a-5p, miR-449c-5p, miR-9-3p, and miR-95-3p) are found exclusively in sMVs and 4 miRNAs (miR-191-3p, miR-197-5p, miR-589-3p, miR-93-3p) are unique to Exos.

In another analysis, we examined miRNAs enriched in the four EV subtypes relative to their respective parental CLs (Fig 3D). It can be seen that 13 miRNAs are commonly enriched in all four EV subtypes. Interestingly, only one miRNA (*miR-7641*) is specifically enriched in both SW620 EV subtypes. Also, there are 7 EV-enriched miRNAs common to exosomes released by the two cell lines, including the only SW480-CL up-regulated miRNA (*miR-210-5p*). Enrichment of *miR-210-3p* (mature miRNA of *miR-210*) is common to SW480-sMVs, SW620-sMVs and SW620-Exos.

### Low-abundance cellular SW480/SW620 miRNAs are significantly enriched in EVs

A salient finding in this study was the significant enrichment of many low-abundance cellular miRNAs (<5 TPM) in secreted EVs. In some cases, these non-detectable cellular miRNAs were found in EVs at levels in excess of 1,000 TPM (S4 Fig and S9 Table). In the case of SW480 cells, 35 non-detectable cellular miRNAs were significantly enriched in SW480-derived EVs (9 exclusively found in sMVs, 7 in Exos and 19 common to both EVs). For SW620 cells, 73 non-detectable cellular miRNAs were found to be enriched in EVs from these cells (55 in sMVs, 4 in Exos and 14 common to both EV subtypes). Further examination of these data reveals striking miRNA distribution patterns. For example, 7 miRNAs non-detectable in SW480-CL (miR-1224-*5p*, *miR-125b-5p*, *miR-7641*, *miR-99a-5p*, *miR-1266-5p*, *miR-194-3p*, and *miR-125b-2-3p*), but enriched in SW480-derived EVs (log2FC in the range 1.36 to 7.94), were found to be up-regulated in SW620-CL (log2FC in the range 1.25 to 4.80); of these, *miR-125b-5p*, *miR-7641* and *miR-99a-5p* are also enriched in SW620 EVs (relative to SW620-CL). Another 7 miRNAs that are highly abundant in SW480-CL yet non-detectable in SW620-CL (*miR-6716-3p*, *miR-26a-1-3p*, *miR-203b-3p*, *miR-891a-5p*, *miR-143-3p*, *miR-371a-5p*, and *miR-509-3p*) are significantly enriched in SW620-derived EVs (log2FC in the range 1.03 to 5.40).

### Correlation of miRNA profiles of SW480/SW620 cells and derived EVs with colorectal normal/tumour tissues

**Fig 4:**
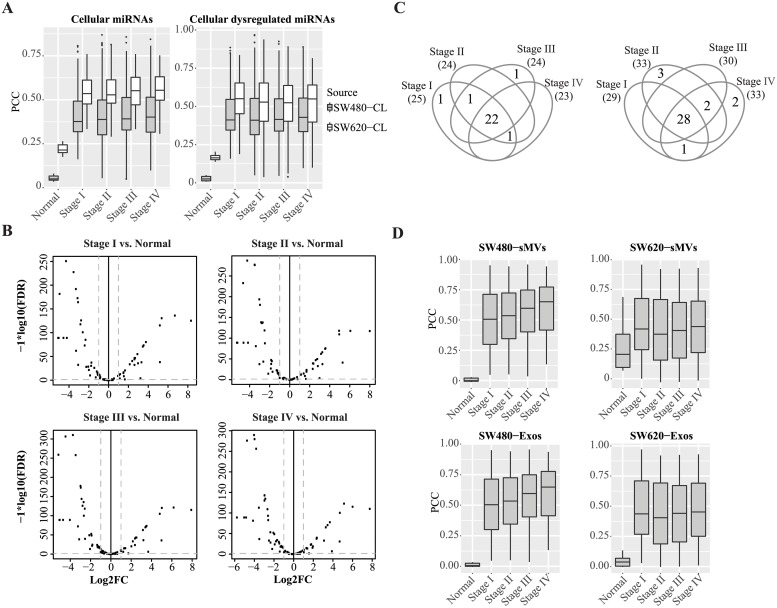
Correlation of miRNAs in SW480/SW620 cell lines and their secreted EVs with miRNAs expressed in non-tumoral colon tissues and CRC tumours. **A**, Pearson correlation coefficients of SW480/SW620 cellular miRNAs and dysregulated cellular miRNAs with miRNA expression profiles of normal colon tissue (11 samples) and 594 tumor samples from different pathological stages; tissue samples were obtained from TCGA. **B**, Volcano plot for comparing 97 EV-enriched miRNAs (from this study) in different CRC tumor stages with normal colon tissue; x-axis: log2FC, y-axis: -log10 (FDR). The vertical dash lines represent the cut-off levels of log2FCs <-1 (down-regulated miRNAs in tumours, relative to normal colon tissue) or >1 (up-regulated miRNAs in tumours, relative to normal colon tissues), respectfully. The horizontal dash line shows the cut-off for FDR <0.05. **C**, Venn diagrams of the number of up-regulated (left) /down-regulated (right) miRNAs identified in CRC tumours of different pathological stages. **D**, Pearson correlation coefficients of SW480/SW620-EV miRNA profiles with miRNA expression profile of different stage colon tumours and normal colon samples.

To investigate the relationship of SW480/SW620 cellular miRNA expression patterns and those reported for primary CRC tumours, we performed a correlation analysis of our cell line miRNA data with CRC tumour miRNA profiles obtained from The Cancer Genome Atlas, TCGA [33]–594 tumours representing 11 normal colon tissues and 103 CRC stage I tumours, 223 stage II, 179 stage III, and 89 stage IV tumours. First, we examined correlations between SW480-CL and SW620-CL and CRC tumours based on 292 and 315 miRNAs, respectively, observed in these cell lines. The mean Pearson correlation coefficients (PCC) for SW480-CL range from 0.05 (normal colon tissue) to 0.41 (for CRC tumours) and, in the case of SW620-CL 0.22 (for normal tissue) to 0.55 for tumours. Based on these correlation coefficients, the miRNA profiles for SW480-CL can be used to predict 592/594 CRC tumours and SW620-CL miRNA profiles, 564/594 (94.9%) of CRC tumours. These predictions improve marginally if the correlations are analysed using the 134 dysregulated miRNAs observed in SW480-/SW620-CLs (see Fig 4A, Table 2)–for example, 593/594 and 572/594 (96.3%) for SW480-/SW620-CLs, respectively. Correlations between SW480/SW620 cell lines and CRC tumours are further illustrated in Fig 4A.

**Table 2:** Correlation of SW480/SW620 miRNA profiles with normal colon tissue and CRC tumors of different pathological stages.

	Normal colon tissue	Stage I CRC tumors	Stage II CRC tumors	Stage III CRC tumors	Stage IV CRC tumors
SW480-CL^a^	0.034~0.080 (0.054)	0.162~0.808 (0.408)	0.054~0.870 (0.410)	0.050~0.858 (0.412)	0.098~0.845 (0.421)
SW620-CL^a^	0.175~0.265 (0.219)	0.333~0.760 (0.539)	0.290~0.815 (0.543)	0.333~0.760 (0.549)	0.307~0.753 (0.559)
SW480-CL^b^	0.007~0.051 (0.027)	0.158~0.886 (0.447)	0.049~0.969 (0.443)	0.045~0.926 (0.441)	0.096~0.890 (0.453)
SW620-CL^b^	0.138~0.204 (0.167)	0.188~0.836 (0.546)	0.038~0.938 (0.526)	0.040~0.893 (0.514)	0.099~0.816 (0.527)
SW480-sMVs^c^	-0.012~0.028 (0.006)	0.047~0.952 (0.497)	0.052~0.943 (0.521)	0.035~0.959 (0.568)	0.134~0.943 (0.591)
SW480-Exos^c^	-0.006~0.035 (0.011)	0.046~0.950 (0.497)	0.050~0.940 (0.521)	0.036~0.956 (0.568)	0.133~0.936 (0.592)
SW620-sMVs^c^	0.068~0.686 (0.246)	-0.000~0.958 (0.444)	-0.030~0.921 (0.410)	-0.027~0.924 (0.415)	-0.016~0.930 (0.431)
SW620-Exos^c^	-0.004~0.134 (0.045)	0.028~0.970 (0.473)	-0.003~0.920 (0.440)	0.000~0.925 (0.443)	0.010~0.930 (0.461)

^a^PCC values are calculated using miRNAs identified in SW480-CL or SW620-CL; values in brackets are mean values.

^b^PCC values are calculated using miRNAs dysregulated in SW620-CL relative to SW480-CL; values in brackets are mean values.

^c^PCC values are calculated using the 97 miRNAs significantly enriched in the EVs compared to the cell lysates; values in brackets are mean values.

Next, in line with the recognition that functional miRNAs in EVs are potential candidates as stable blood-based biomarkers, we performed PCC analyses between SW480/SW620 cell-derived EVs (sMVs and Exos) and CRC tumours. A total of 97 miRNAs significantly enriched in SW480/SW620 cell-secreted EVs (sMVs and Exos) were used for these analyses (see S3–S6 Tables). As shown in the volcano plots in Fig 4B more than half of the EV-enriched miRNAs were dysregulated in different CRC tumour stages compared to normal colon tissue. The number of miRNAs up-regulated and down-regulated revealed in different CRC tumour stages is given in the Venn diagrams shown in Fig 4C. For the EV-enriched miRNAs, we next calculated their PCC values between normal colon tissue and different tumour stage tissues (Table 2). A salient finding of this study is that the mean PCC values between EV miRNA profiles and normal colon tissue miRNA profiles are striking lower than the mean PCC values between cellular miRNA profiles and normal colon tissue miRNA profiles, thereby enhancing CRC tumour prediction accuracy (Fig 4D, Table 2).

### Correlation of miRNA profiles of SW480/SW620 cells and derived EVs with CRC patient serum/plasma exosomal miRNA profiles

**Fig 5:**
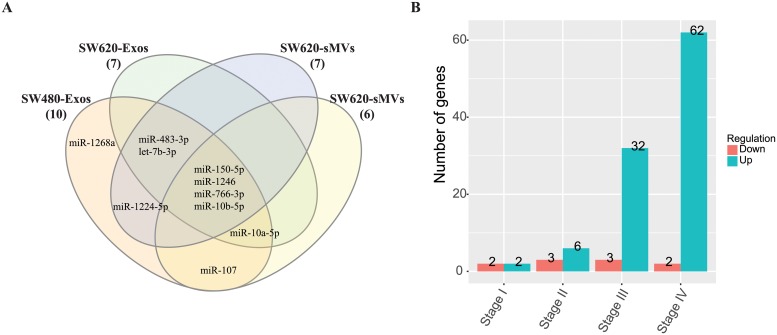
Comparison of miRNA profiles of SW480/SW620 EVs and circulating EVs. A. Venn diagram of miRNAs enriched in SW480/S620 EVs and miRNAs up-regulated in the serum EVs of CRC patients. B. Number of miRNAs enriched in SW480/SW620 EVs that are differentially expressed in the plasma EVs of different CRC stage patients.

To further evaluate the potential of SW480/SW620 EV-enriched miRNAs as CRC biomarkers, we compared the 97 miRNAs with previously reported serum/plasma exosomal miRNA profiles of CRC patients [20, 21]. The serum exosomal miRNA study screened 88 primary CRC patients and 11 HCs using microarray technology and identified 69 miRNAs were up-regulated in CRC patients than healthy controls. Among them, miR-1280 and miR-1308 were removed from miRBase because they are tRNA fragments of a. Of the remaining, 67 miRNAs, 10, 7, 7 and 6 were selectively enriched in the SW480-Exos, SW480-sMVs, SW620-Exos and SW620-sMVs, respectively (Fig 5A). Four miRNAs (miR-150-5p, miR-1246, miR-766-3p and miR-10b-5p) were commonly enriched in the EVs. In addition, we found that miR-1268a was specifically enriched in SW480-Exos and miR-107 was enriched in only SW480-CL released EVs.

Next, we compared the 97 miRNAs against plasma exosomal miRNA profiles of different CRC stage patients (58 stage I, 46 stage II, 28 stage III and 27 stage IV) and HCs (n = 50). Differential expression analysis (fold change > 1.5 or < -1.5, p-value < 0.05 and FDR < 0.05) showed that 4, 9, 35 and 64 miRNAs were differentially expressed in stage I, II, III and IV, respectively, relative to the HCs (Fig 5B, S10 Table). It is clear that in advanced CRC stage patients more miRNAs were dysregulated in the plasma exosomes. Because 7 miRNAs (miR-1266-5p, miR-3620-3p, miR-4640-3p, miR-4758-3p, miR-5096, miR-6720-5p and miR-6840-3p) were expressed less than 5 TPM in the plasma exosomes, we compared the 92 EV-enriched miRNAs with the 84 differentially expressed miRNAs in CRC plasma exosomes (Table 3). It is shown that among the EV-enriched miRNAs only miR-342-3p, commonly enriched in SW480-CL released EVs, was down-regulated in the plasma exosomes of stage III CRC patients (S10 Table). We found that miR-483-5p was the only miRNA enriched in SW480 EVs and up-regulated in the plasma exosomes of stage I CRC patients (Table 3). The up-regulation of miR-483-5p was also found in stage II and IV CRC patients. While miR-1246 was the only miRNA which was abundant in all four EV subtypes and up-regulated in stage II, III and IV CRC patients, which maybe a broad biomarker for CRC cancer. Several EV-enriched miRNAs were found to be up-regulated only in advanced CRC patients, such as let-7b-3p, miR-27a-5p, miR-182-5p, miR-192-5p and miR-486-5p. The Pearson correlation model was not suitable for these two datasets as the linear correlation was very low (< 0.1) between SW480/SW620 EVs and serum/plasma exosomal miRNA profiles.

**Table 3:** Number of EV-enriched miRNAs up-regulated in exosomes isolated from CRC-patient plasma compared to exosomes from healthy individuals*.

CRC stage	SW480-Exos	SW620-Exos	SW480-sMVs	SW620-sMVs
*Stage I*	miR-483-5p		miR-483-5p	
*Stage II*	miR-1246 miR-483-5p	miR-1246	miR-1246 miR-483-5p	miR-1246
*Stage III*	miR-182-5p miR-1246 miR-486-5p miR-27a-5p	miR-486-5p miR-182-5p miR-1246 miR-27a-5p miR-550a-5p	miR-1246 miR-182-5p	miR-1246 miR-182-5p miR-486-5p
*Stage IV*	miR-1246 miR-486-5p let-7b-3p miR-192-5p miR-483-5p	miR-486-5p miR-1246 let-7b-3p miR-550a-5p miR-181a-2-3p	miR-1246 let-7b-3p miR-192-5p miR-483-5p	miR-1246 miR-486-5p

*Data from Ogata-Kawata et al [19].

## Discussion

This study was designed to compare miRNA expression profiles of isogenic CRC cell lines SW480 (from primary carcinoma) and SW620 (from lymph node metastasis) [25] and their secreted EVs (sMVs and Exos), with the aim understanding the role of miRNAs in the aetiology of CRC. Further, we examined the suitability of miRNA profiles of SW480/SW620 cell-line-secreted EVs as clinical indicators for predicting CRC.

First, we compared miRNA expression profiles of SW480 and SW620 cells. This study revealed 292 miRNAs in SW480-CLs and 315 miRNAs in SW620-CLs. Of these, 30 and 53 miRNAs were found to be exclusively expressed in SW480- and SW620-CLs, respectively. Using edgeR, a total of 134 cellular SW480/SW620 miRNAs were found to be significantly dysregulated (log2FC>1 or log2FC<-1, p-value <0.05, FDR <0.05) with 61 SW480-CL miRNAs up-regulated in SW620-CL and 73 miRNAs down-regulated (S2 Table). KEGG pathway analysis of the down-regulated miRNAs identified significant (p<0.0.1) enrichment in “Wnt signalling pathway” (ko04310) terms, while the up-regulated miRNAs were enriched in “TGF-beta signalling pathway” (ko04350) terms. These observations are consistent with the biological role of Wnt/TGF-β signalling (involving genes APC, AXIN1/2, GSK3β, the TCF gene family and TP53) in CRC [34, 35]. For example, dysregulation of Wnt signalling is an early event during CRC initiation [35] and dysregulation of TGF-β signalling pathway plays a critical role in cancer metastasis [36]. According to dbDEMC 2.0 [37], a database of differentially expressed miRNAs in all human cancers, all 134 dysregulated miRNAs identified in our study are listed. While *miR-486-3p* and *miR-561-5p* have been previously shown to be dysregulated in lung and kidney cancers (dbDEMC 2.0), the up-regulation of *miR-486-3p* and down-regulation of *miR-561-5p* in SW620-CL is the first report, to our knowledge, of their dysregulation during CRC progression. Interestingly, the known CRC metastasis suppressor miRNAs *miR-141* [38] and *miR-203* [39] were found to be significantly down-regulated in SW620-CL compared to SW480-CL. Notably, another two anti-oncomiRNAs, *miR-34c* and *miR-342*, that operate upstream of Wnt were found to be highly expressed in SW480-CL but not detectable in SW620-CL using a <5 TPM threshold cut off. It has been previously reported that the expression of these two miRNAs in CRC primary tumours is silenced by CpG island methylation of their encoding DNA sequences [40, 41] suggesting that depletion of *miR-34c* and *miR-342* levels might contribute to CRC metastasis. Other miRNAs down-regulated in SW620-CL (compared to SW480-CL) such as *miR-125a* and *miR-10a* also have been reported to suppress metastasis of lung cancer [42] and gastric cancer [43]. Of those oncomiRNAs up-regulated in SW620-CL (relative to SW480-CL), *miR-181a*, *miR17~92a* cluster (*miR-19a/b* and *miR-92a*, *miR-17*, *miR-18a* and *miR-20a*) and *miR-1246* are implicated in CRC development [44–49]. Paradoxically, in addition to these oncomiRNAs, we see several anti-oncomiRNAs up-regulated in SW620-CL (e.g., *miR-100*, *miR-142*, *miR-192* and *miR-194*); controversially, these miRNAs are frequently down-regulated in CRC [50–52].

Second, and in line with the observation that EVs released from cells can localise to blood [19, 53–55], we characterized the miRNA profiles of SW480/SW620-derived sMV and Exos EV subtypes. For both CRC cell lines, many cellular oncomiRNAs /anti-oncomiRNAs (e.g., *miR-10b*, *miR-100*, *miR-192*, *miR-200c*, and *miR-222*) were selectively sorted into EVs (Fig 3A and 3B). In all, 186 miRNAs were detected in SW480/SW620-derived EV subtypes, many being in common and several exclusively distributing to each EV subtype (Fig 3C). Similar to our previous miRNA profiling of three CRC LIM1863 cell line-derived EV populations [23], each SW480/SW620 cell-derived EV subtype has a distinct miRNA portrait (Fig 3C) containing many potential candidate biomarkers that can be explored for CRC diagnosis and pursued in future clinical studies.

Third, our intriguing observation that miRNAs non-detectable (i.e., <5 TPM) in SW480/SW620-CLs can be selectively sorted into sMVs and Exos and actively packaged in high concentration (e.g., > 1000 TPM) (S4 Fig and S9 Table) represents, to the best of our knowledge, the first documentation of such a paradigm. Whether the elimination of certain clusters of cellular miRNAs (e.g., miRNAs that modulate Wnt/β-catenin and TGF-β activities) via EVs represents a vehicle for lowering the cellular concentration of these miRNA clusters, thereby impacting on biological function, is not known. Counterpoint to this speculation is whether these same EV-containing miRNAs cleared from the parental cell act synergistically to regulate Wnt/β-catenin and TGF-β activities in recipient cell(s) upon EV uptake. Needless to say, the underlying mechanism of this phenomenon warrants investigation. We have shown that miRNA expression patterns vary between parental SW480/SW620 cells and their cognate EVs. These data suggest that specific structural motifs must exist to sort distinct miRNAs into EVs. We therefore performed a motif search in all 371 EV miRNAs identified in this study (S3–S6 Tables, derived from 334 miRNA precursors) that might serve as targeting signals. No global enrichment of specific sequences or motifs was found, including GGAG which is reported to be recognized by hnRNP A2B1 [56]; this motif was identified in 108 miRNAs. However, an interesting finding was a 52.7% enrichment (176/334) of the pre-miRNA motif CUGU reported for the RNA-binding protein adenamotous polyposis coli (APC) [57] which also plays a pivotal role in the canonical Wnt/β-catenin signalling pathway [58].

Fourth, the observation that *miR-7641* is not only up-regulated in SW620-CL, compared to SW480-CL, but also significantly enriched (log2FC in the range 6.1 to 11.5) in both SW480 and SW620 EVs (Fig 3D, S3–S6 Tables) is striking. There are two possible regions in the human genome that can encode *miR-7641*, one the 5S rRNA gene (RNA5SP387), the other in an intergenic region. It has been previously reported that *miR-7641* is a potential biomarker of aging [59] while, in another report, it has been shown to modulate the expression of CXCL1 during endothelial differentiation derived from human embryonic stem cells [60]. According to the dbDEMC database, miR-7641 was down-regulated in blood samples from pancreatic, biliary tract, colon, gastric, oesophageal and liver cancer patients. While the biological function of *miR-7641* remains largely unknown, its significant enrichment (log2FC in the range 6.1–11.5) in SW480- and SW620-EVs makes it an attractive candidate for the diagnosis and prognosis of CRC.

Finally, because of the stability of EV-miRNAs in the circulatory system [61], we reasoned that miRNA profiles of EVs (sMVs and Exos) from the SW480/SW620 cell lines might contain attractive candidate biomarkers for the diagnosis of CRC. Given that there are many differences in mRNA profiling of cancer cell lines and tumours [62, 63] due to the absence of stromal and immune components *in vitro*, we undertook a correlation analysis between miRNA profiles of SW480/SW620 cell line-derived EVs and CRC tumour tissues to gauge the extent that EV miRNA signatures mirror pathological changes in CRC tumours. Our study shows that the mean PCC values between EV miRNA signatures and normal colon miRNA signatures are significantly lower than the mean PCC values between cellular miRNA signatures and normal colon tissue (presumably, due to the striking enrichment in EVs of miRNAs non-detectable in SW480-/SW620-CLs), resulting in improved tumour prediction accuracy using EV miRNA signatures. Our results show that the Pearson correlation coefficient approach based on EV (sMVs and Exos) miRNA signatures has a high sensitivity (94.9%) and specificity (100%) to predict CRC tumours of different stages listed in the TCGA dataset. More CRC cell line-derived EV profiles are required to assess the correlation of miRNA expression in tumours according to pathological stage. Also, some EV-enriched miRNAs are up-regulated in serum/plasma exosomes from CRC patients, compared to HCs, especially in advanced CRC pathological stages (Fig 5). This not only indicates that EVs can transfer regulatory miRNAs that promote cancer progression but also shows EV-containing miRNAs have demonstrated potential CRC biomarkers. The low linear correlation of miRNA profiles in cell-derived EVs and circulating EVs may be due to the different technologies used for miRNA profiling and/or the predicting model needs to be improved.

In summary, there is emerging recognition that EV miRNAs represent promising candidates as stable biomarkers for CRC detection [20], and as prognostic biomarkers for recurrence of CRC [64]. Moreover, the unique properties of EVs provide a potential tool for the development of drug delivery systems for cancer therapy [65, 66]. It is anticipated that many of the EV-associated miRNAs identified in the present study might represent attractive candidate biomarkers for the diagnosis of CRC and be useful prognostic indicators of this disease.

## Supporting information

S1 Fig Total RNA profile analysis using Agilent 2100 Bioanalyzer. (PDF)

S2 Fig qRT-PCR. (PDF)

S3 Fig IPA analysis for cellular dysregulated miRNAs. (PDF)

S4 Fig Venn diagram of low-abundance cellular miRNAs in the EVs. (PDF)

S1 Table 345 miRNAs identified in SW480 and SW620 cell lysates (> 5 TPM). (XLSX)

S2 Table Differentially expressed miRNAs between SW480 and SW620 cell lysates. (XLSX)

S3 Table miRNAs detected in sMVs of SW480. (XLSX)

S4 Table miRNAs detected in exosomes of SW480. (XLSX)

S5 Table miRNAs detected in sMVs of SW620. (XLSX)

S6 Table miRNAs detected in exosomes of SW620. (XLSX)

S7 Table miRNAs detected in all EV subtypes of two CRC cell lines. (XLSX)

S8 Table miRNAs specifically distributed into the EVs released by SW620 cells. (XLSX)

S9 Table miRNAs > 5 TPM in EVs but < 5 TPM in the cell lysates. (XLSX)

S10 Table Differentially expressed genes in plasma exosomes of CRC patients. (XLSX)

## Abbreviations

3’-UTR: 3’untranslated region
CEA: carcinoembryonic antigen
CLs: cell lysates
CRC: colorectal cancer
EV: extracellular vesicles
Exos: exosomes
miRNA: microRNA
qRT-PCR: quantitative real-time PCR
SEER: Surveillance, Epidemiology, and End Results
sMVs: shed microvesicles
TCGA: The Cancer Genome Atlas
TMUGS: Tumour Marker Utility Grading System
TPM: transcripts per million reads
