# Supplementary figures and images for: Distinct shed microvesicle and exosome microRNA signatures reveal diagnostic markers for colorectal cancer
Source: PLoS One. 2019 Jan 4;14(1):e0210003. doi: 10.1371/journal.pone.0210003 (PMC6319712; doi:10.1371/journal.pone.0210003)

SW480-CL-R1

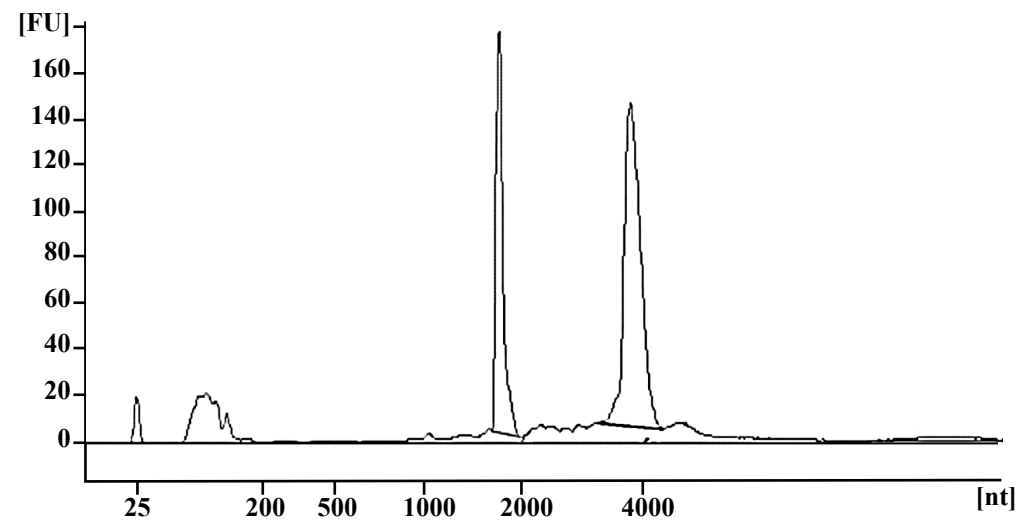

SW480-sMVs-R1

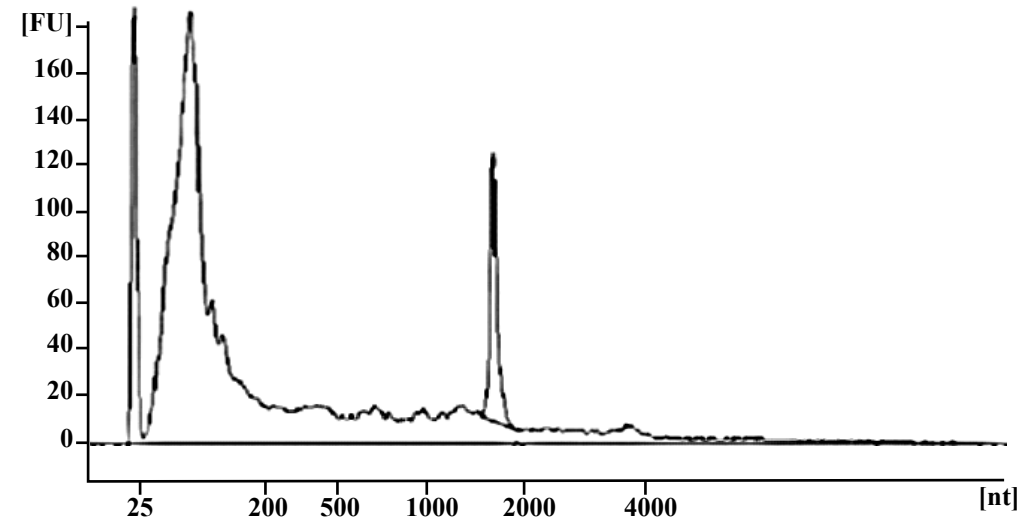

SW480-Exos-R1

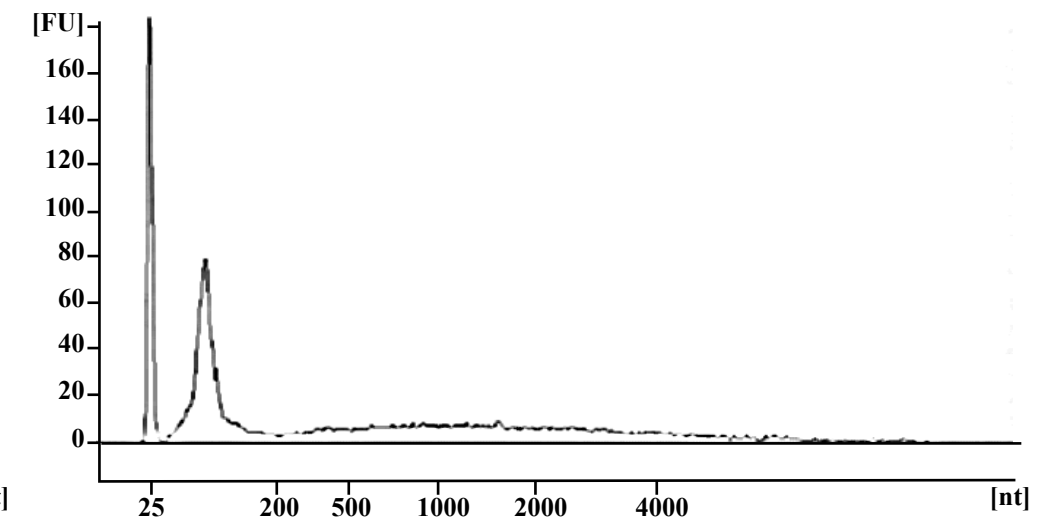

SW480-CL-R2

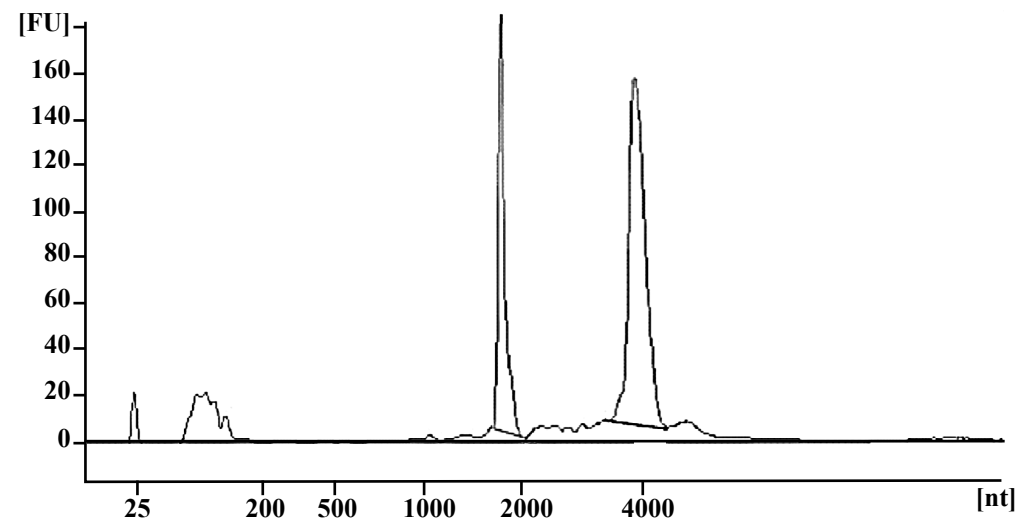

SW480-sMVs-R2

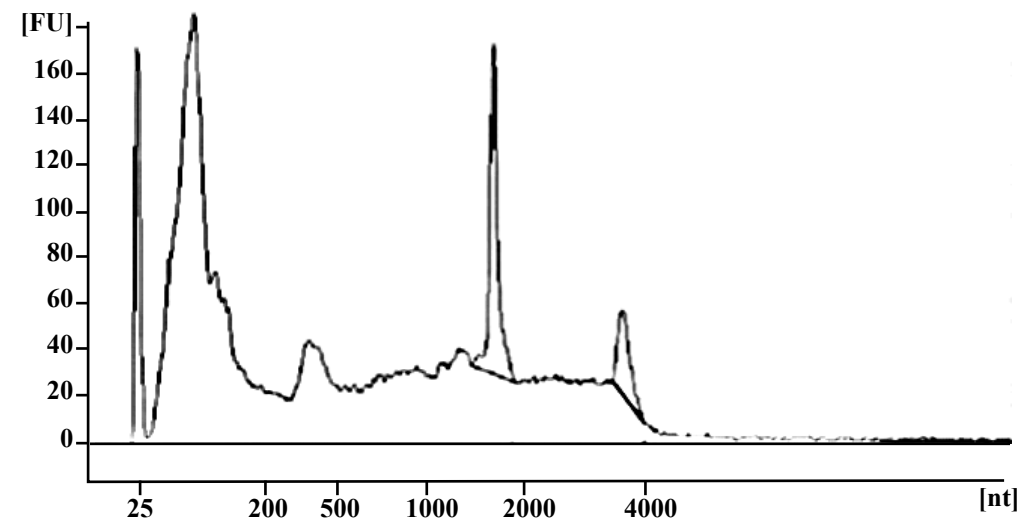

SW480-Exos-R2

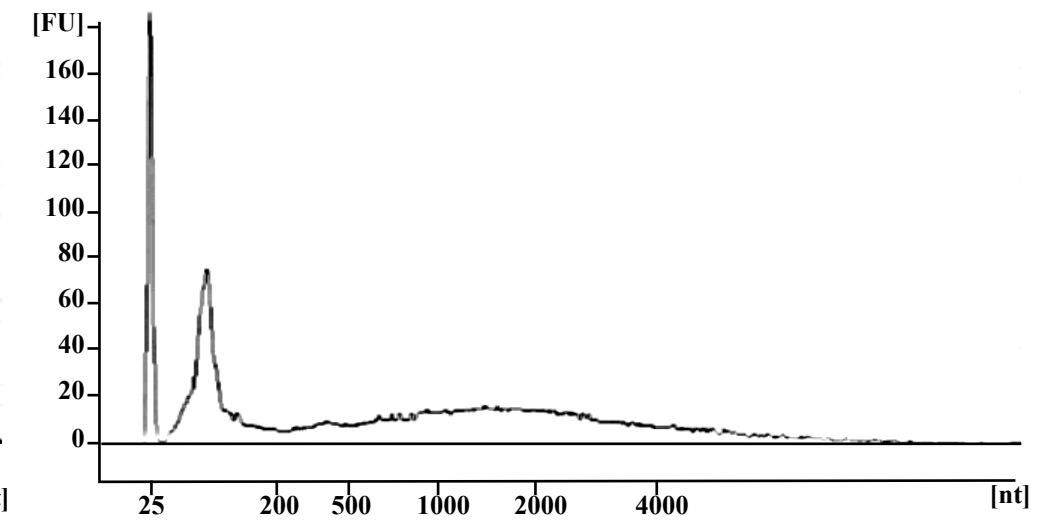

SW620-CL-R1

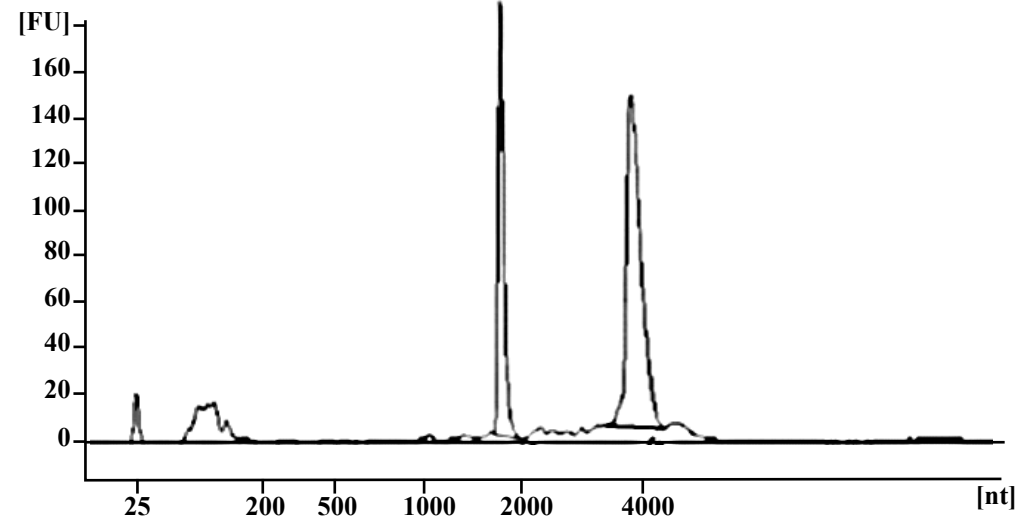

SW620-sMVs-R1

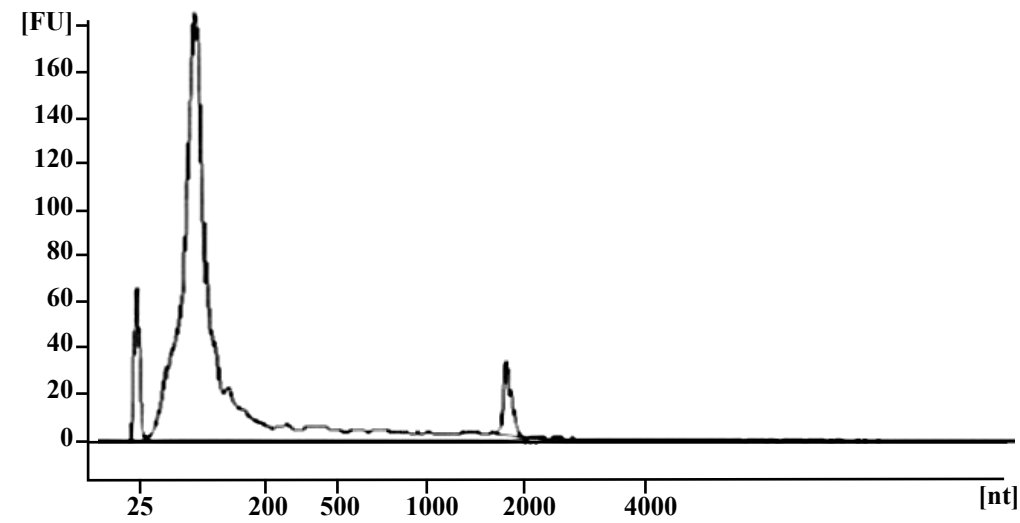

SW620-Exos-R1

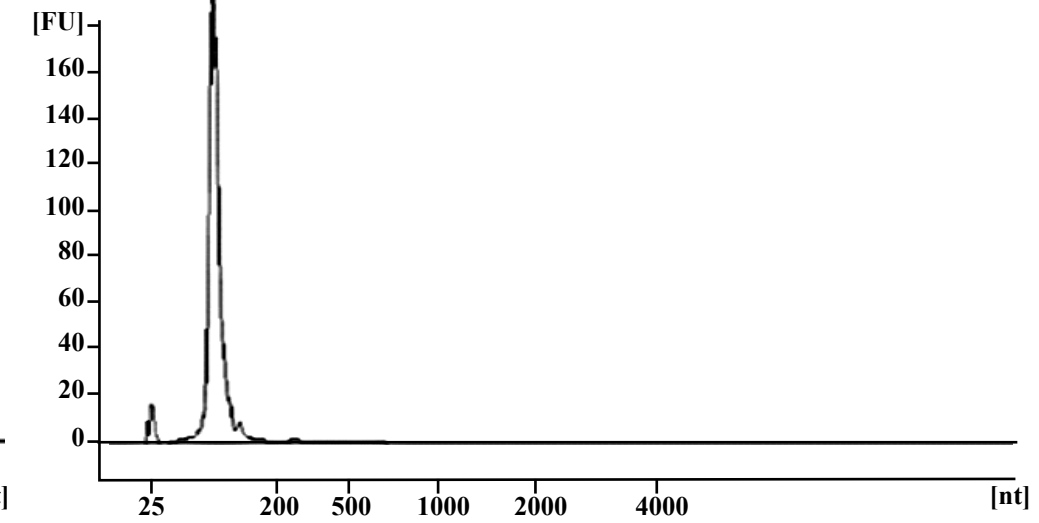

SW620-CL-R2

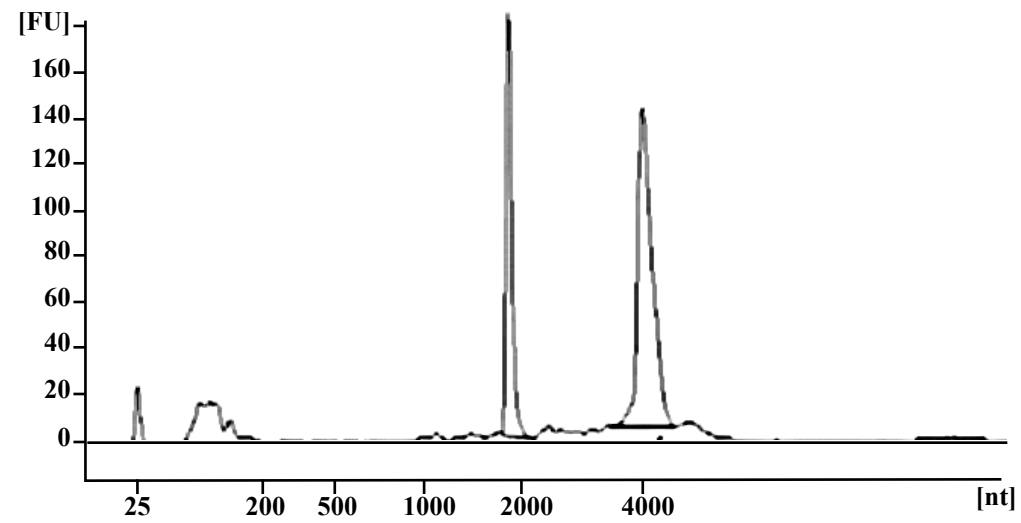

SW620-sMVs-R2

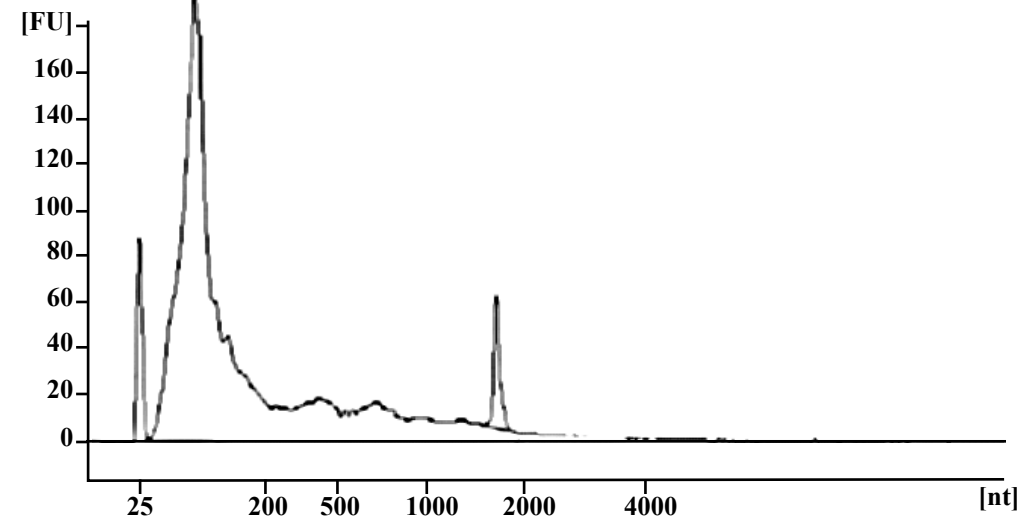

SW620-Exos-R2

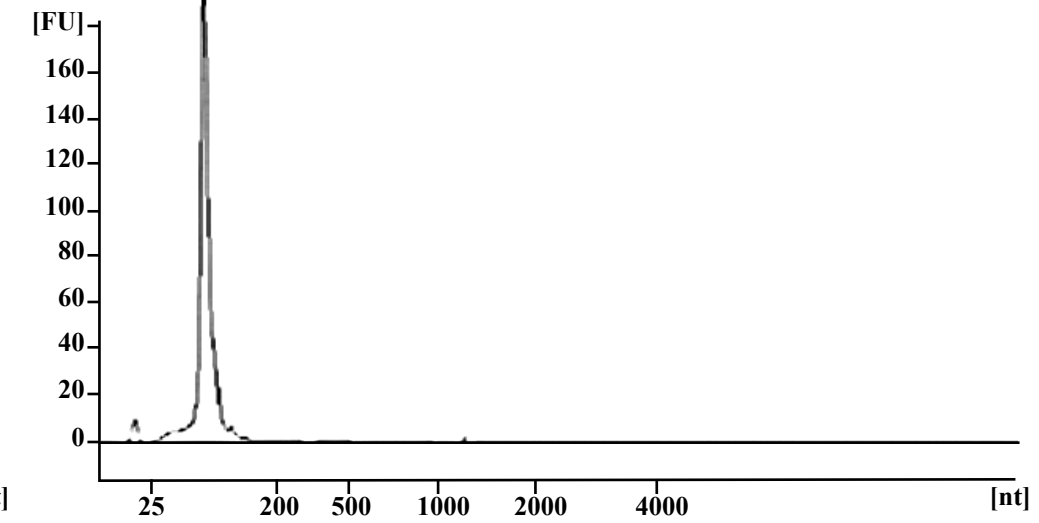

Supplement: S1 Fig — (PDF) [file pone.0210003.s001.pdf]

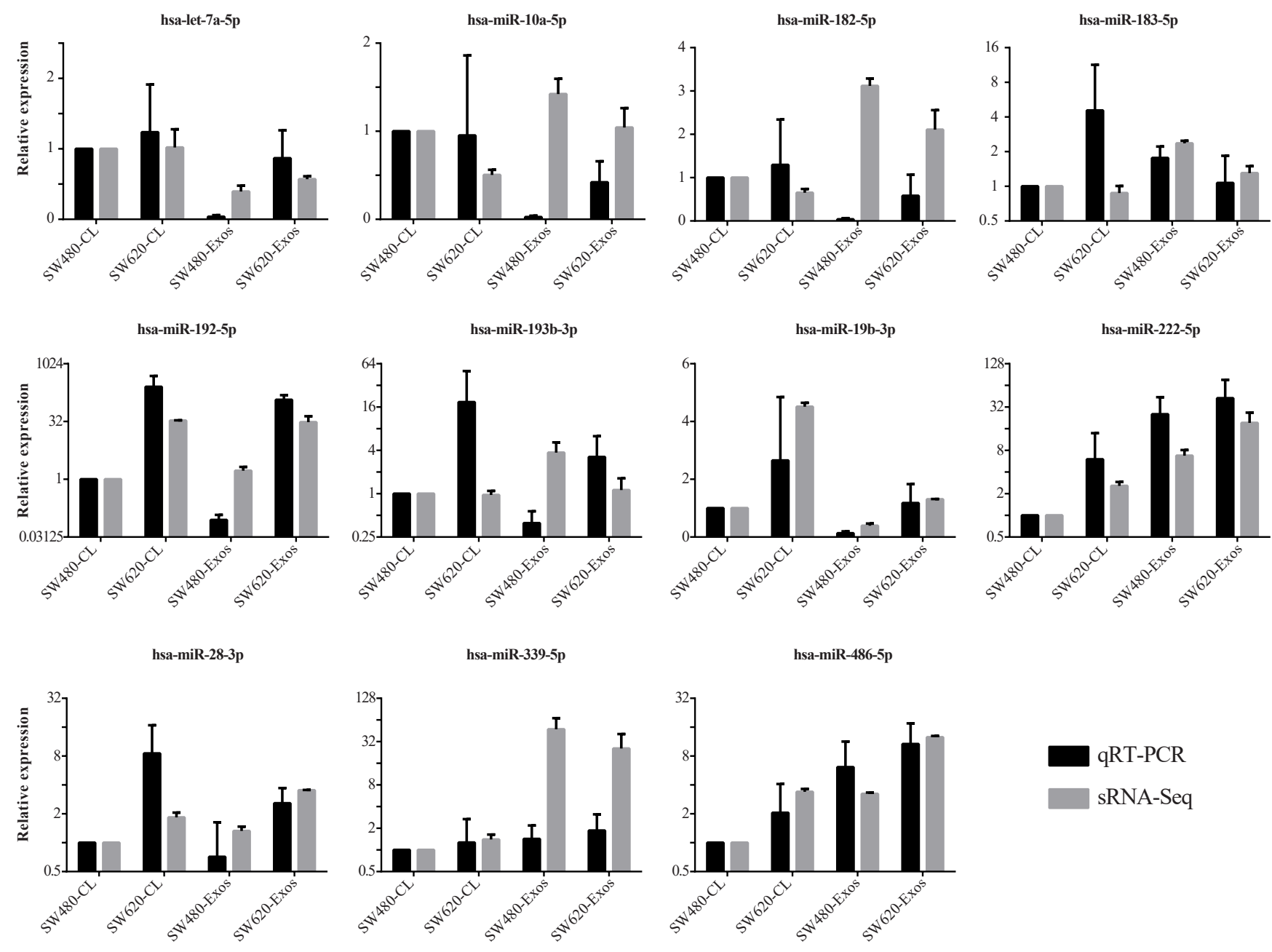

Supplement: S2 Fig — (PDF) [file pone.0210003.s002.pdf]

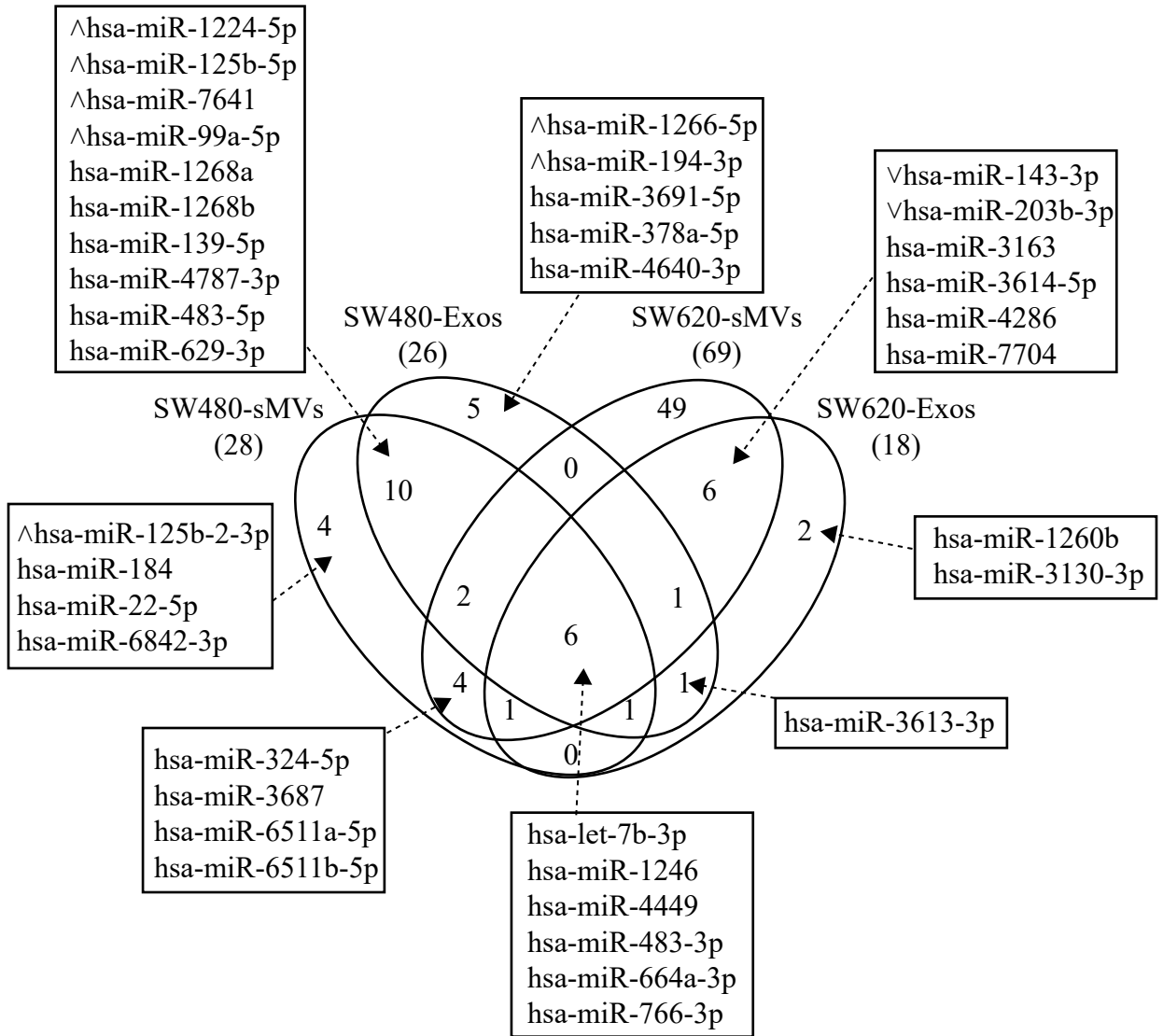

Supplement: S4 Fig — (PDF) [file pone.0210003.s004.pdf]
