# Supplementary material for: Distinct shed microvesicle and exosome microRNA signatures reveal diagnostic markers for colorectal cancer
Source: PLoS One. 2019 Jan 4;14(1):e0210003. doi: 10.1371/journal.pone.0210003 (PMC6319712; doi:10.1371/journal.pone.0210003)
Supplement: S3 Fig — (PDF) [file pone.0210003.s003.pdf]

A

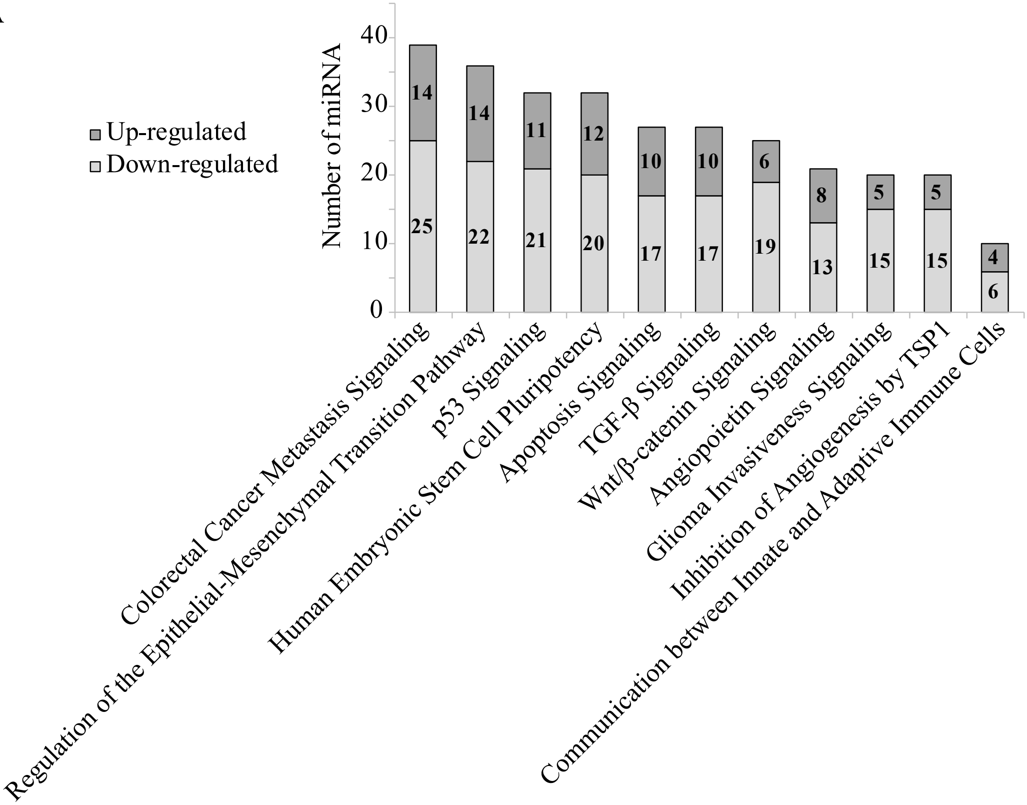

B

miRNA	Colorectal Cancer Metastasis Signaling	p53 Signaling	Wnt/β-catenin Signaling
hsa-miR-125b-5p	SMO,TP53	CASP6,CDKN2A,PERP,TP53	CDH5,CDKN2A,SMO,TP53
hsa-miR-17-5p	CCND1,JAK1,MMP3,STAT3,TGFBR2, TLR7,TNF,VEGFA	BCL2,CCND1,CDKN1A, E2F1,PTEN,RB1,TP63	BMPR2,CCND1,TGFBR2
hsa-miR-181a-5p	KRAS,MMP14	BCL2	NLK
hsa-miR-19a-3p	CCND1	CCND1,PTEN,THBS1	BMPR2,CCND1
hsa-miR-100-5p	FGFR3	FGFR3	
hsa-miR-1271-5p	ADCY6,IRS1	IRS1	
hsa-miR-142-3p*	ADCY9,BCL2L1	BCL2L1	
hsa-miR-135a-5p	APC,JAC2		APC
hsa-miR-199b-3p*	PTGS2		CD44
hsa-miR-125b-1-3p*	TNF		
hsa-miR-140-5p	SMAD3,VEGFA		
hsa-miR-18a-3p*	KRAS		
hsa-miR-301b-3p	SMAD4		
hsa-miR-375	JAK2		
hsa-miR-153-3p		BCL2	
hsa-miR-18a-5p		BCL2	
hsa-miR-199b-5p		HIF1A,SIRT1	
hsa-miR-486-5p		PTEN	
